# Clinical Applications of Platelet-Rich Plasma in Patellar Tendinopathy

**DOI:** 10.1155/2014/249498

**Published:** 2014-07-21

**Authors:** D. U. Jeong, C.-R. Lee, J. H. Lee, J. Pak, L.-W. Kang, B. C. Jeong, S. H. Lee

**Affiliations:** ^1^School of Medicine, Korea University College of Medicine, 73 Inchon-ro, Seongbuk-gu, Seoul 136-705, Republic of Korea; ^2^National Leading Research Laboratory, Department of Biological Sciences, Myongji University, 116 Myongji-ro, Yongin, Gyeonggi-do 449-728, Republic of Korea; ^3^Stems Medical Clinic, 32-3 Chungdam-dong, Gangnam-gu, Seoul 135-950, Republic of Korea; ^4^Department of Biological Sciences, Konkuk University, 1 Hwayang-dong, Gwangjin-gu, Seoul 143-701, Republic of Korea

## Abstract

Platelet-rich plasma (PRP), a blood derivative with high concentrations of platelets, has been found to have high levels of autologous growth factors (GFs), such as transforming growth factor-*β* (TGF-*β*), platelet-derived growth factor (PDGF), fibroblastic growth factor (FGF), vascular endothelial growth factor (VEGF), and epidermal growth factor (EGF). These GFs and other biological active proteins of PRP can promote tissue healing through the regulation of fibrosis and angiogenesis. Moreover, PRP is considered to be safe due to its autologous nature and long-term usage without any reported major complications. Therefore, PRP therapy could be an option in treating overused tendon damage such as chronic tendinopathy. Here, we present a systematic review highlighting the clinical effectiveness of PRP injection therapy in patellar tendinopathy, which is a major cause of athletes to retire from their respective careers.

## 1. Introduction

Platelet-rich plasma (PRP) is prepared by centrifuging anticoagulated whole blood obtained by phlebotomy. Therefore, it contains a hyperphysiological concentration of autologous platelets, 3–8 times the concentration of platelets in whole blood [1]. However, the exact definition of PRP has not been determined in terms of the concentration of platelet, and most published reports differ on PRP concentrations [2].

Platelets are nonnucleated cytoplasmic bodies derived from megakaryocyte precursors. They play a pivotal role in hemostasis and wound healing via the formation of fibrin clots [1, 3]. Therefore, increasing platelet concentration in compromised (or injured) tissue may result in an exponential release of diverse bioactive factors and, subsequently, enhance the healing process [1]. PRP therapy is considered safe, because it has an autologous nature and long-term clinical effects without any reported major side effects [1, 4–6]. In addition to the safety, the easy availability of PRP leads to application in clinical and surgical settings. However, the efficacy of PRP therapy has not yet been clearly defined [1, 4–6]. This systematic review article will demonstrate the properties of PRP and its application in clinical therapy (especially focusing on patellar tendinopathy).

When PRP injection occurs, highly concentrated platelets are activated. As a result, there is an exponential increase in numerous GFs (Table 1) at the sight of injection [1]. These various GFs include insulin-like growth factor (IGF-1), transforming growth factor (TGF-*β*), platelet-derived growth factor (PDGF), vascular endothelial growth factor (VEGF), fibroblast growth factor (FGF), platelet-derived angiogenic factor (PDAF), and platelet-derived endothelial growth factor (PDEGF) [1, 2]. Hepatocyte growth factor (HGF), epidermal growth factor (EGF), cytokines, chemokines, and metabolites also appear to be involved [1, 2, 7]. Bioactive molecules that facilitate various components of healing exist in higher concentrations in platelets than in native blood [8].

Expression of a variety of GFs has a central role in the healing processes of tissues, including those of tendon and ligament [9, 10]. The tendon healing process progresses in three phases. At the beginning, the inflammatory phase occurs for 24 hours. Neutrophils and macrophages play a role in producing chemotactic and vasoactive factors. The proliferative phase follows the inflammatory phase and is dominated by the synthesis of collagen type III and granulation tissue [11]. The last stage is the remodeling phase, which begins approximately six weeks later with a decrease in cellular and vascular content and an increase in collagen type I content [9, 12].

Clinical studies using cultured human tenocytes have shown that PRP, in the form of platelet-rich clot releasate (PRCR), the active releasate of PRP, stimulates differentiation of human tendon stem cells into active tenocytes with high proliferation rates and total collagen production [1, 13]. Therefore, PRP has a number of potential beneficial properties, such as the release of GFs that could restart healing in the injured tissues [8].

## 2. Methods

**Figure 1:**
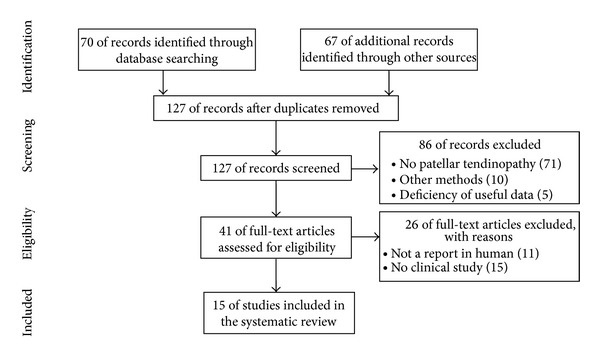
Literature selection process (PRISMA flow diagram).

We used the Preferred Reporting Items for Systematic Review and Meta-Analysis (PRISMA) in our review [14] (Figure 1). We conducted a systematic literature search in the following databases: Medline via PubMed and the Cochrane Library. Additionally, we also searched on the following Web sites: National Institute for Health and Care Excellence (http://www.nice.org.uk), Canadian Agency for Drugs and Technologies in Health (http://www.cadth.ca), Current Controlled Trials (http://www.controlled-trials.com), and BioMed Central (http://www.biomedcentral.com). We used keywords as search terms. We combined terms for selected indications (platelet-rich plasma, patellar, tendinopathy, tendinosis, tendonitis, tendinitis, and tendon). The literature search included all studies published in English between 2000 and 2014. We identified 127 references after removing duplicates. We independently assessed full-text articles for inclusion in our review. The criteria for inclusion of studies in our review encompassed all clinical trials of PRP injection conducted on humans with patellar tendinopathy. After discarding 15 review articles, we identified 15 clinical trials (two randomized controlled trial (RCT) studies, six nonrandomized controlled trial (non-RCT) studies, two prospective case-series study, three case studies, and two retrospective studies).

## 3. PRP Treatments in Patellar Tendinopathy

PRP is a bioactive component of whole blood, which is now being widely tested in different fields of medicine [4]. The use of PRP to favor tendon healing has been advocated only relatively recently [10, 15, 16]. Many researchers have been encouraged to investigate the effects of PRP injections in tendinopathy and to measure the outcomes.

Clinicians are increasingly using the term “tendinopathy” to refer to tendon disorders [17]. The term is currently accepted to indicate an overuse pathological condition in and around tendon. It refers to degenerative changes with lack of inflammatory features (“tendinosis”) or inflammatory process (“tendonitis” or “tendinitis”) [18]. At histopathological examination, tendinopathy is a failed healing response. In addition to an increase in noncollagenous matrix and neovascularization, it is characterized by haphazard proliferation of tenocytes and disruption and altered organization of collagen fibers [18, 19]. Tendinopathy is overuse injuries frequently associated with sports. It usually occurs in major tendons, such as the Achilles, patellar, rotator cuff, and forearm extensor tendons. This review mainly focuses on the effectiveness of PRP, especially for a refractory chronic patellar tendinopathy.

### 3.1. PRP in Patellar Tendinopathy

PRP may offer opportunities in aiding regeneration of tissue with low healing potential as in patellar tendinopathy [4, 20–22]. A complex regulation of several GFs stimulates the expression of procollagen types I and III, improves mechanical properties, and promotes tendon cell proliferation and tendon healing [4, 20]. Since patellar tendinopathy is one of major injuries that cause athletes to retire from their field, we focused on the evaluation of clinical studies documenting the potential of PRP treatment for patellar tendinopathy.

Through a systematic literature search, we found 15 clinical studies about the efficacy of PRP treatment on patellar tendinopathy: two RCT studies, six non-RCT studies, two prospective case-series study, three case studies, and two retrospective studies. The main features of these studies were summarized in Table 2. Most subjects were athletes in various sports and their ages ranged from 18 to 73 years. Most patients had not improved with various previous other treatments.

### 3.2. RCT by Vetrano et al

Extracorporeal shock wave therapy (ESWT) and PRP injections seem to be a safe and promising part of the rehabilitation program for jumper's knee, although, given current knowledge, it is impossible to recommend a specific treatment protocol. Both treatments share the same disputes: lack of hard evidence through randomized clinical trial and no standardized treatment protocols [23]. Vetrano et al. [23] compared two autologous PRP injections versus three sessions of ESWT, through randomized controlled trial. The PRP group showed significantly better improvement than the ESWT group in the Victorian Institute of Sports Assessment-Patellar questionnaire (VISA-P) and visual analog scale for pain (VAS) scores at 6- and 12-month follow-up and in modified Blanzina scale score at 12-month follow-up [23]. Therefore, this report shows that therapeutic injections of PRP lead to better midterm clinical results compared with focused ESWT in the treatment of jumper's knee in athletes [23]. The explanation for better results in the PRP group may be related to a multifaceted mechanism of action involving platelet action as well as injection-related effects [23–25]. Additionally, the high expectations of patients about this new technology may have a great influence especially in sports medicine. The principal limitations of this study are the small number of patients enrolled, the lack of a placebo control group, and follow-up assessment through qualitative outcome measures in the absence of clinical and instrumental quantitative assessments (e.g., color ultrasonography, magnetic resonance imaging (MRI)) [23]. In addition, although the assessment was blinded, there was no way to blind the patients to the treatment. Therefore, it is possible that their awareness of the treatment modality may have had some effect on their perception of their response to the treatment and the results may be specific to the specific formulation of PRP and the specific ESWT protocol used in the study [23].

### 3.3. RCT by Dragoo et al

A second RCT studyis recently published. The study compared a regimen of eccentric exercises combined with either ultrasound-guided PRP injection or ultrasound-guided dry needling alone in the treatment of patellar tendinopathy [26]. The PRP group showed significantly better improvement than the dry needling group in VISA-P score at 12 weeks. However, at 26-week follow-up, the difference between the PRP and dry needling groups dissipated in all assessed scores, such as VISA-P, Tegner, VAS, and short form-12 (SF-12) scores. In other words, at 26-week follow-up, the dry needling group had also made clinically and statistically significant improvements on VISA-P, Tegner, Lysholm, and VAS scores [26]. A previous result also showed that dry needling and injection of autologous blood for patellar tendinopathy show promise as an alternative treatment for this chronic condition [25]. Although dry needling does not introduce additional volume into the tendon, it can stimulate a healing response within the tendon by initiating bleeding [26]. Therefore, additional studies could compare the effect of PRP injection versus dry needling, to better understand the importance of injection composition or injection in itself. A limitation of this study is that anatomic tendon changes using ultrasound or MRI were not measured.

### 3.4. Non-RCT by Volpi et al

Volpi et al. [21] provided preliminary proof about the efficacy of PRP injections for the treatment of chronic patellar tendinopathy. The VISA-P and MRI were used to evaluate the clinical outcomes of eight high-level athletes. The results represented a 91% average improvement in VISA-P score, and MRI images at the final follow-up demonstrated a noticeable reduction in irregularity of the affected tendon compared with preinjection images for 80% of the treated tendons.

### 3.5. Non-RCT by Kon et al

Kon et al. [4] aimed to explore PRP application to treating chronic patellar tendinopathy, by gathering and assessing the number, timing, severity, duration, and resolution of related adverse events occurring among study participants before and after treatment. They also evaluated the results, to determine the feasibility, safety, and potential of this application. Tegner, EuroQol-visual analogue scale (EQ-VAS), and short form-36 (SF-36) questionnaires were used to assess the clinical outcome. A statistically significant improvement in all scores was observed up to six months after the treatment [4]. Follow-up revealed that the postprocedure protocol markedly influenced the results: participants who did not follow the rehabilitation programme achieved poorer results [4]. The results suggest that this PRP application may be safely used for the treatment of chronic patellar tendinopathy, by aiding the regeneration of tissue that otherwise has low healing potential [4]. However, this study lacked a control group and had a low number of patients treated. Furthermore, direct data (such as a histopathological examination) confirming the regeneration of damaged patellar tendon was not shown.

### 3.6. Non-RCT by Filardo et al

Filardo et al. [22] used a non-RCT to evaluate the efficacy of multiple PRP injections on the healing of chronic refractory patellar tendinopathy after previous classical treatments had failed. The preparation and injection of platelet concentrate and postinjection phase used in this study were similar to the therapeutic procedures used by Kon et al. [4]. Outcome measures included Tegner, EQ-VAS, and pain level. A statistically significant improvement in all scores was observed at the end of the PRP injections in patients with chronic refractory patellar tendinopathy and a further improvement was noted at six months, after physiotherapy was added [22]. The result showed significantly better improvement in sports activity level in the PRP group than in the control group. In other words, patients with a long history (much longer with respect to that of the control group) of chronic refractory jumper's knee, who had previous failed nonsurgical or even surgical treatments, were able, through a combination of multiple PRP injections and physiotherapy, to achieve the same results obtainable in less severe cases. As patients were subjected to PRP and physiotherapy simultaneously, the fact that the relative importance and the real contributions of two therapies to the therapeutic outcome were indistinguishable represents the limitation of this study. The small number of patients treated and the lack of randomization (not usable in this case due to the predetermined different selection criteria) are also weak points of this study.

### 3.7. Non-RCT by Gosens et al

Gosens et al. [27] aimed to evaluate the outcome of patients with patellar tendinopathy treated with PRP injections, and they examined whether certain characteristics, such as activity level or previous treatment, affected the results. Clinical evaluations were made by VISA-P and VAS, assessing pain in activities of daily life (ADL), during work and sports, before and after treatment with PRP. After PRP treatment, patients with patellar tendinopathy showed a statistically significant improvement [27]. There was a significant difference between those that had chronic tendinopathy without previous failing therapies and those with chronic tendinopathy of the same duration but with previous failing treatments. Although all patients of two groups significantly improved on the VAS scales, patients with previous failing treatments showed a smaller healing potential on VISA-P than patients without previous failing therapies. The main limitation of this study is also the fact that it is a nonrandomized and noncontrolled study [27].

### 3.8. Non-RCT by Ferrero et al

Ferrero et al. [28] aimed to evaluate the effectiveness of ultrasound-guided autologous PRP injections in patellar and Achilles tendinopathy. Clinical (using VISA score) and ultrasound evaluation of 28 patellar tendons (4 bilateral) in 24 patients who underwent ultrasound-guided PRP injection were performed after 20 days and 6 months after the injection. In this study, the 6-month follow-up showed that ultrasound-guided PRP injection improved symptoms and tendon structure [28]. The significantly improved VISA scores at the 6-month follow-up were consistent with results previously obtained by other reports [4, 28]. Moreover, the fact that tendon thickness and hypoechoic areas were reduced may be a sign of tendon regeneration, as collagen fibers were more closely packed like in normal tendons. Finally, the increased power Doppler signal, both at the 20-day and 6-month follow-up, is a sign of an induced vascular response needed to improve tendon regeneration [28]. The authors concluded that PRP injection in patellar and Achilles tendinopathy results in a significant and lasting improvement of clinical symptoms and leads to recovery of the tendon matrix potentially helping to prevent degenerative lesions [28]. The main limitation of this study is the lack of a control group.

### 3.9. Non-RCT by Filardo et al

To evaluate the therapeutic effects of multiple PRP injections on the healing of chronic refractory patellar tendinopathy, Filardo et al. [29] assessed the quality and duration of the clinical improvement up to a midterm (a mean 48 months) follow-up in a cohort group. They also evaluated the changes in neovascularization level induced by PRP injections and its correlation with the clinical findings. They treated 43 patients with multiple injections of PRP and evaluated the patients by Blanzina, VISA-P, EQ-VAS for general health, and Tegner scores. The results documented were good and stable with the VISA-P score. The same trend was confirmed by other scores used. To a midterm follow-up, PRP injections provided a good clinical outcome. This report is the only study examining PRP effect at midterm follow-up. The ultrasound measurements showed that tendon thickness and neovascularization level gradually decrease over time, despite an initial increase after the injection cycle [29]. No correlation ultrasonographic and clinical finding could be found. Therefore, the authors emphasize that there is a need to evaluate it carefully when managing a tendinopathic condition and to rely mainly on the clinical condition until new studies will provide more insights into the significance of imaging findings [29]. The study has some limitations, such as the imaging evaluation performed only in some of the patients at different follow-ups and the lack of a randomized control group.

### 3.10. Prospective Case Series by van Ark et al

A physical therapy program performs an important role after PRP injection treatment in patellar tendinopathy patients, because a mechanical loading is needed after this injection [30]. van Ark et al. [31] (prospective case-series study) reported the first results of a combination treatment of PRP injection followed by a well-described physical therapy program. Five of the six tendons show an improvement of at least 30 points on the VISA-P after 26 weeks and four of the five patients indicated that they would positively recommend this treatment to family or friends with the same injury. In accordance with a pilot study on PRP by Kon et al. [4], the only patient who did not show improvement after treatment was the one with the lowest self-reported program compliance. This study proposed a combination treatment of an injection with PRP followed by a physical therapy program, because a previous eccentric exercise physical therapy program and other treatments alone did not result in positive effects [31]. Limitations of this study are the small number of participants, the lack of a control group, and the heterogeneity of the participants. It is important to state that due to several limitations of this case series, no well-grounded statements can be made on the effectiveness of the treatment.

### 3.11. Prospective Case Series by Charousset et al

Charousset et al. [32] reported the prospective case results of 28 patients with patellar tendinopathy treated by PRP injection. At the 2-year follow-up, the average preprocedure VISA-P, VAS, and Lysholm scores of 28 patients improved from 39 to 94, 7 to 0.8, and 60 to 96, respectively [32]. Twenty-one of 28 patients recovered to their presymptom sporting level at three months after the PRP injection. MRI scan exhibited complete recovery of 16 patients to normal structural integrity of the tendon and significant improved structural integrity of the tendon in all other patients. The major limitations of this study are the absence of a control group and the variation in the cellular content of PRP in terms of GFs, platelet concentrations, and platelet activation. The ideal protocol of PRP preparation has yet to be determined.

### 3.12. Case Study by Brown and Sivan

Three case studies were performed. Brown and Sivan [33] treated a 36-year-old active cricketer presenting with a 9-month history of right knee pain. He had to discontinue cricket because of the severity of his pain. The patient's symptoms did not improve despite a 9-month trial of conservative treatment. By using a single point of entry, 3 mL of PRP was injected under ultrasound guidance into multiple regions of the tendinopathic proximal patellar tendon. The outcome from the PRP injection was superior to the conventional physical therapy program, and the benefit has been maintained, even eight months after the procedure.

### 3.13. Case Study by Rowan and Drouin

A 23-year-old female athlete was managed for bilateral patellar tendinopathy with a combination of traditional therapeutic interventions as well as a PRP injection. This athlete returned to preinjury level of competition six months after injection [34]. This case report was of a high-level athlete treated more aggressively to allow for an earlier return to competition and may not be the ideal course of treatment for the general population. This report emphasizes the possible benefits of adding PRP injections as a complementary therapy along with manual therapy, pain-relieving modalities, shock wave therapy, and eccentric exercises. Considering the limited value of a single case report with the absence of a control group, further research is warranted to more conclusively determine the best course of therapy for patellar tendinopathy [34].

### 3.14. Case Study by Scollon-Grieve and Malanga

An 18-year-old male competitive high school lacrosse player was managed for patellar tendinopathy with a PRP injection. Two months after receiving the PRP injection, he returned to full activity and competitive collegiate lacrosse participation [35]. The author emphasizes that the PRP injection is a safe and promising alternative for patients with patellar tendinopathy. This report also has the limited value of a single case report with the absence of a control group.

### 3.15. Retrospective Study by Mautner et al

There are two retrospective reports investigating outcomes of patients treated with ultrasound-guided PRP injections at multiple academic institutions for chronic tendinopathies, including patellar tendinopathy. Mautner et al. [17] (a multicenter, retrospective review; Table 2) aimed to determine whether ultrasound-guided PRP injections are an effective treatment for chronic tendinopathies. The primary outcome measurement was the perceived improvement in symptoms at least six months after the PRP injection(s). This perception was quantified using a Likert scale: “not at all,” “slightly,” “moderately,” “mostly,” and “completely.” Secondary outcome measurements were the following: perceived change in VAS before and after the procedure, functional pain after the procedure using the Nirschl Pain Phase Scale for overuse injuries, and overall satisfaction with the PRP procedure (quantified with the following Likert scale) [36]. No significant difference was found between the patients who answered the survey at one year or less after the PRP procedure and those who answered more than one year after the procedure, thus refuting the argument that the observed improvements were simply due to spontaneous resolution of symptoms. The authors studied the response of multiple tendons treated throughout the body and determined overall improvement in symptoms. As a result of this trial, in patellar tendon group, 78% of patients reported more than 50% improvement in VAS and more than half of their patients reported at least a moderate improvement in symptoms. Therefore, the authors concluded that the majority of patients reported a moderate improvement in pain symptoms among patients with patellar tendinopathy, as with patients with pain in other tendons. The limitation of this study is that the response rate in the survey was 55%. In addition, some patients did not follow up long term with their physician and thus may have benefited from additional treatments. This study also required that a rehabilitation program be completed but did not standardize the specific protocol.

### 3.16. Retrospective Study by Dallaudière et al

A second retrospective report was recently reported [37]. Dallaudière et al. also aimed to assess the efficacy and tolerance of intratendinous injection of PRP to treat tendinopathy in a large group of patients. This study included 408 patients (250 patients with tendinopathy in the upper limb and 158 patients with tendinopathy in the lower limb). Among 408 patients, 41 patients had patellar tendinopathy. Independent of age, gender, and type of tendinopathy, Western Ontario and McMaster Universities Osteoarthritis Index (WOMAC) scores and residual US size of lesions were significantly improved at 6-week follow-up after the ultrasound-guided injection of PRP. The average WOMAC scores of 41 patients with patellar tendinopathy improved from 38 to 16 at the 6-week follow-up and more improved (6 scores) at 32-month follow-up. No clinical complication was reported during follow-up [37]. This study demonstrates that the ultrasound-guided injection of PRP allows rapid healing of tendon with good tolerance. The limitations of this study are a lack of histologic assessment and the absence of a control group. Additionally, the authors did not describe whether or not a rehabilitation program after the PRP injection is performed.

## 4. Discussions

PRP has the potential to recruit numerous GFs necessary for wound healing. Due to its ability to regulate fibrosis and angiogenesis, it can be applied on tendon injury. Several animal studies showed that the application of PRP enhances and accelerates the patellar tendon healing process through activation of numerous GFs [1, 38, 39], especially by overexpression of IGF-1 [40]. Based on animal model studies, there are 15 clinical reports to treat patellar tendinopathy. These clinical studies include two RCT studies, six non-RCT studies, two prospective case-series study, three case studies, and two retrospective studies. All reports suggest that PRP injection is an effective treatment for patellar tendinopathy. Clinical evaluations have been carried out using various evaluation tools, including VISA-P, VISA-A, VAS, EQ-VAS, SF-36 questionnaires, Tegner, pain level, NS, (modified) Blanzina, Likert scale, functional pain, overall satisfaction, ultrasound examination, and MRI images. A statistically meaningfully improvement in most evaluation scores was observed after PRP treatment. It is noteworthy that PRP treatment is not only effective in short-term follow-up (at 6 months), but good and stable results were also obtained in longer follow-up, such as 12 months or 4 years. Therefore, the clinical injection of PRP seems to be more preferable to a long-term treatment, due to its long-term persistence.

In spite of these recent clinical reports about the effect of PRP for the treatment of patellar tendinopathy, many limitations of PRP studies make it hard to draw clear conclusions concerning the effectiveness of PRP treatment from these results. First of all, most studies did not use a control group with the same population characteristics as the treatment group. The small number of patients treated and the lack of a randomized control group should be also improved. Case studies for a long-term period (more than two years) can assist the verification process of PRP effect on patellar tendinopathy. Despite the therapeutic applications of PRP in patellar tendinopathy as well as in many other injured sites (such as Achilles, rotator cuff, and forearm extensor tendons), little information is available for the mechanism of its action. Understanding the exact working mechanism of PRP can enhance the efficacy of this clinical treatment. Lastly, the effects of rehabilitation protocols after PRP treatment must also be established. Animal model studies showed that mechanical stimulation should initiate as soon as possible after PRP injection because PRP influences especially the early phases of regeneration [30, 41]. A recent study proposed a simple and efficient 6-week rehabilitation program based on submaximal eccentric reeducation to add to PRP infiltrations in case of patellar tendinopathy [42].

PRP has an autologous nature and no critical complications or side effects have been reported, suggesting that this treatment could be considered safe. Nevertheless, up to now, the standard application protocol and the definition of PRP have not been established clearly. In the clinical application using PRP, significant differences in platelet concentration or the overall cell types contained in PRP could be happened [43]. These variations are strictly linked to the procedures employed [43]. There are two main preparation methods used in clinical practice: the use of a laboratory centrifuge or a density gradient cell separator. In the use of a laboratory centrifuge, various parameters, such as speed, timing, number of centrifugation, and technician-dependent reproducibility, could affect contents of the final PRP product. A density gradient cell separator is a closed-circuit device that allows PRP preparation without excessive manipulation of the blood [44]. However, because a large number of these devices with its own features were available, it is impossible to obtain the same PRP product in all clinical trials. In some cases, the PRP products could contain leukocytes and residual blood cells, besides platelets and plasmas [43, 44]. As leukocytes can release matrix metalloproteases and reactive oxygen species capable of damaging articular tissues, some people insisted that leukocytes can induce inflammatory effect in the tendon [44]. Really, a recent animal model study showed that leukocyte-rich PRP causes a significantly greater acute inflammatory response than leukocyte-poor PRP at 5 days after injection [45], indicating that the difference of PRP preparation can affect the host's cellular response. Therefore, more detailed protocols for PRP treatment, such as standard preparation method, injection dosage, and injection method, will increase the quality of PRP research.

Even though several conservative treatments, such as eccentric training and ESWT, have been proposed for tendinopathy, very few of them are supported by randomized controlled trials [46]. Moreover, no single treatment has been proven to result in a consistent, near-complete recovery in all patients [20, 47–49]. The therapeutic exercise shows improvement of clinical symptoms when used alone, but it shows greater improvement when combined with PRP injection [31]. ESWT is useful in patellar tendinopathy only for improving subjective symptoms, but does not show an actual clinical improvement in objective parameters [50, 51]. Vetrano et al. [23] compared two PRP injections versus three sessions of ESWT, through an RCT study. Therapeutic injections of PRP lead to better midterm clinical results compared with focused ESWT in the treatment of jumper's knee in athletes.

All injection therapies (steroid, aprotinin, high-volume, autologous blood injection, and cell therapy) can relieve symptoms of patients [20]. Aprotinin injection has a lasting beneficial effect for patellar tendinopathy patients [52, 53], but, in very few cases, side effects of allergy were reported in the tendon trials [54, 55]. The role of steroids in the management of tendinopathy is also controversial [20]. Although a steroid injection positively affected the short-term follow-up of tendinopathy, it failed in long-term follow-up improvement [56]. And the side effect of steroid injection, such as tendon rupture, has been reported [57–59]. Compared with steroids and ESWT, PRP injection therapy is shown to be more effective in long-term follow-up [60]. Crisp et al. [61] evaluated a novel conservative management modality for patellar tendinopathy. They hypothesized that disruption of neovascularization could be achieved by mechanical means, namely, by injecting large volumes of fluid (high-volume injection) at the interface between the posterior aspect of the paratenon of the patellar tendon and the area from where the neovessels penetrate the tendinopathic lesion, the so-called Hoffa's body. They found that the injection of a large volume of mixtures combined with bupivacaine, hydrocortisone, and normal saline triggered significant improvements in VAS and VISA-P scores, suggesting that the injected volumes, regardless of the injected contents, can affect improvement of patellar tendinopathy through mechanical disruption of neoneurovascularization. It is hard to compare high-volume injection with PRP injection and conclude definitely which therapeutic option shows better outcome, because of the differences between the participants' characteristics. Nevertheless, when follow-up periods and the overall VISA-P improvement between high-volume injection therapy and PRP injection therapy were compared, PRP showed better improvement. One possible hypothesis is that the steroid having poor clinical outcomes on patellar tendinopathy is commonly used in the high-volume injection therapy, to prevent inflammatory reactions induced by injection of large quantities of foreign materials.

Unlike the steroid, ESWT, or the high-volume injection, autologous blood injection was significantly effective on patellar tendinopathy [25]. However, as autologous blood injection and dry needling are combined with physiotherapy as part of their treatment protocol, the effect of the autologous blood injection alone cannot be estimated. To the best of our knowledge, no studies have compared the injections with autologous GFs or with PRP. The rationale for autologous blood injection closely resembles the previously described rationale for PRP. Autologous preparations that are rich in GFs induce cell proliferation and promote synthesis of angiogenic factors during the healing process [20]. Some believe, however, that GFs work in a dose-dependent manner, and hence a more concentrated source of GFs, such as that provided by PRP, is needed for the technique to be beneficial [12, 62]. In a recent study at competition horses with overuse musculoskeletal injuries (suspensory ligament desmopathy and superficial flexor tendinopathy), significantly faster recovery was observed in cases of PRP with high concentrations of platelets [63], supporting the belief that GFs work in a dose-dependent manner. Recently, Dragoo et al. showed that the dry needling had also made clinically and statistically significant improvements of patellar tendinopathy on VISA, Tegner, Lysholm, and VAS scores [26]. Although dry needling does not introduce additional volume into the tendon, it can stimulate a healing response within the tendon by initiating bleeding [26]. Therefore, additional studies could compare the effect of PRP injection versus dry needling or the autologous blood injection, to better understand the importance of injection composition and injection volume.

Bone marrow mononuclear cells (BM-MNCs) are pluripotential cells and are believed to play an important role in connective tissue repair such as tendon, ligament, bone, and cartilage [64]. Unlike other injection therapies introduced above, two clinical trials using cell therapy in patients with chronic patellar tendinopathy showed significant improvements in Tegner score (Pascual-Garrido et al. [64]) and VISA-P (Clarke et at. [65]). Although those studies are conducted individually and controls of procedures are different, according to the results, the cell therapy could be considered as a potential therapy for those with refractory chronic patellar tendinopathy. So far, as the number of studies is low and only few high-quality studies for the treatment of jumper's knee for human are available, it is hard to draw firm conclusions on the effectiveness of the cell therapy and compare it with PRP. Moreover, the exact role of implanted stem cells on tendon healing remains uncertain [64]. It is not clear at what time point inoculation should be considered, the number of applications needed, and whether to combine it with PRP or not. PRP treatment, in the form of PRP-clot releasate (PRCR), promotes differentiation of tendon stem cell (TSC) into active tenocytes exhibiting high proliferation rates and collagen production capability [13]. PRP has also been successfully used as a cell culture additive to facilitate growth and differentiation of autologous mesenchymal stem cells (MSCs) [66–70]. So, PRP in combination with cell therapy could be promising in the patellar tendinopathy. However, further critical review and rigorous clinical studies are required to determine the real effectiveness of this combination therapy in the management of chronic patellar tendinopathy.

In conclusion, injection therapy of PRP is effective for the treatment of patellar tendinopathy and has the promising potential to restore patients to their activities of daily living, work, and sports. However, through the present research, it is hard to draw a clear conclusion for the effectiveness of PRP treatment on patellar tendinopathy. More precise clinical researches are required and the standard application protocols, including standard preparation method, injection dosage, and injection method, must also be established. In addition, PRP treatment in combination with the cell therapy could more efficiently cure patients with the patellar tendinopathy. Thus, more high-quality clinical studies on combination therapy are certainly required.

## Figures and Tables

**Table 1:** GFs in PRP∗.

Growth factor	Function
EGF	Cellular proliferation
	Differentiation of epithelial cells

FGF	Stimulates angiogenesis
	Cellular migration
	Stimulates the proliferation of capillary endothelial cells
	Production of granulation tissue

HGF	Stimulation of hepatocyte proliferation and liver tissue regeneration
	Stimulates angiogenesis
	Mitogen for endothelial cells
	Antifibrotic

IGF-1	Proliferation of myoblasts and fibroblasts
	Stimulation of protein synthesis
	Mediator in growth and repair of skeletal muscle
	Enhances bone formation by proliferation and differentiation of osteoblasts
	Enhances collagen and matrix synthesis

PDAF	Induces vascularization by stimulating vascular endothelial cells

PDEGF	Stimulates the proliferation of keratinocytes and dermal fibroblasts

PDGF	Macrophage activation
	Stimulates angiogenesis
	Fibroblast chemotaxis and proliferative activity
	Attracts stem cells and white blood cells
	Enhances collagen synthesis
	Contributes to tissue remodeling
	Enhances the proliferation of bone cells

TGF-*β*	Enhances the proliferative activity of fibroblasts
	Stimulates biosynthesis of type 1 collagen and fibronectin
	Induces deposition of bone matrix
	Inhibits osteoclast formation and bone resorption
	Regulation in balance between fibrosis and myocyte regeneration
	Control of angiogenesis and fibrosis
	Immunosuppressant during inflammatory phase

VEGF	Stimulates angiogenesis
	Migration and mitosis of endothelial cells
	Creation of blood vessel lumen
	Chemotactic for macrophages and granulocytes
	Vasodilation

EGF: epidermal growth factor; FGF: fibroblast growth factor; HGF: hepatocyte growth factor; IGF-1: insulin-like growth factor-1; PDAF: platelet-derived angiogenic factor; PDEGF: platelet-derived endothelial growth factor; PDGF: platelet-derived growth factor; TGF-*β*: transforming growth factor-*β*; VEGF: vascular endothelial growth factor.

∗Data from [1, 2, 7, 8].

**Table 2:** Clinical studies on PRP treatments for patellar tendinopathy.

Study (yr)	Intervention treatment (per group)	Study type	Number of subjects (total/study group; sex)	Subject characteristic (age; symptoms' duration)	Previous therapy	Concurrent treatment	Follow-up	Outcome measures	Results	Authors' conclusion
Vetrano et al. (2013) [23]	G1: 2x USG PRP (2 mL) injections every 1 wk G2: 3 sessions of focused ESWT (2.400 impulses at 0.17–0.25 mJ/mm^2^ per session)	RCT	G1: 23; 20 M/3 F G2: 23; 17 M/6 F	G1: 26.9 ± 9.1 yr; mean 18.9 mo G2: 26.8 ± 8.5 yr; mean 17.6 mo	Various treatments without any success	G1, G2: standardized stretching, muscle strengthening protocol; gradual return to sports activities (after 4 wk)	2 mo; 6 mo; 12 mo	VISA-P, VAS, modified Blanzina scale	G1 showed significantly better improvement than the G2 in VISA-P, VAS scores (6, 12 mo FU) and in modified Blanzina scale score (12 mo FU)	Therapeutic injections of PRP lead to better midterm clinical results compared with focused ESWT in the treatment of jumper's knee in athletes

Dragoo et al. (2014) [26]	G1: USG PRP (6 mL) + 0.25% bupivacaine (3 mL) + 1 : 100,000 epinephrine injections; 10x MP G2: USG 0.25% bupivacaine (3 mL) + 1 : 100,000 epinephrine injections; 10x MP	RCT	G1: 9; 8 M/1 F G2: 12; 12 M/0 F	G1: 28 ± 8 yr G2: 40 ± 14 yr	Various treatments without any success	G1, G2: physical therapy twice per week; standardized additional exercises at home	3 wk; 6 wk; 9 wk; 12 wk; ≥6 mo	VISA-P; Tegner; Lysholm; VAS; SF-12	G1 showed significantly better improvement than the G2 at 12 wk (*P* = 0.02), but the difference between two groups was not significant at ≥26 wk (*P* = 0.66)	PRP injection accelerates the recovery from patellar tendinopathy relative to USG dry needling, but the apparent benefit of PRP dissipates over time

Volpi et al. (2007) [21]	G1: 0.5 mL of local anaesthetic (lidocaine) injected; 1x USG (3 mL) PRP injected G2: no control group	Non-RCT; prospective cohort study	8/8; 7 M/1 F (3 bilateral)	26.6 (21–41) yr; at least 1 yr	Various treatments without any success	Rest, walking (1st 7 d); stretching exercises, exercise bike, walking in water, light swim (7–21 d); eccentric quadriceps training, concentric strengthening (after 5 wk); muscular strengthening, jogging (after 7 wk); normal sport activities (after 12 wk)	7 d; 30 d; 60 d; 120 d	VISA-P; MRI	Statistically significant improvement in VISA-P score for 7 out of 8 patients treated	Valid therapeutic option (PRP)

Kon et al. (2009) [4]	G1: PRP injections (3x) were administered every 15 d without USG; before the injection, 10% of CaCl_2_ was added to the PRP unit (5 mL with ca. 6.8 million platelets) to activate platelets; 4–6x MP G2: no control group	Non-RCT; prospective cohort study	20/20; 20 M (7 bilateral)	25.5 (18–47) yr; 20.7 (3–60) mo	Various treatments without any success	Rest (between 1st and 2nd injection); stretching exercises and mild activities (after 2nd injection); stretching exercises and mild activities (after 3rd injection); normal sport activities (after 1 mo)	ET; 6 mo	Tegner; EQ-VAS; SF-36 questionnaires	Statistically significant improvements in all scores	Safe application, aiding the regeneration of tissue with low healing potential; long-term RCT needed

Filardo et al. (2010) [22]	G1: PRP injections (3x) were administered every 15 d without USG; before the injection, 10% of CaCl_2_ was added to the PRP unit (5 mL with ca. 6.5 million platelets) to activate platelets; 4–6x MP G2: no injection	Non-RCT	31/15; 31 M (5 bilateral)	G1: 28.8 ± 8.5 yr; 24.1 ± 19.9 mo G2: 25.5 ± 9.2 yr; 8.4 ± 4.1 mo	G1: various treatments without any success G2: without treatment (at least 2 mo), primarily physiotherapy protocol only	Rest (between 1st and 2nd injection); stretching exercises and mild activities (after 2nd injection); stretching exercises and mild activities (after 3rd injection); normal sport activities (after 1 mo)	ET; 6 mo	Tegner; EQ-VAS; pain level	Statistically significant improvements in all scores	PRP can be useful for the treatment of chronic patellar tendinopathy, even in difficult cases with refractory tendinopathy (only physiotherapy approach had failed)

Gosens et al. (2012) [27]	G1 and G2: 1 mL of PRP + bupivacaine HCl 0.5% + epinephrine injection (1st injection); remaining PRP + bupivacaine HCl 0.5% + epinephrine (ca. 4 mL) injected (2nd injection)	Non-RCT; prospective cohort study	36/36; 23 M/13 F	30.9 ± 12.6 yr; 40.3 ± 28.4 mo	G1: 14, various treatments without any success G2: 22, without treatment	Rest (1st 24 hr); standardized stretching protocol (after 24 hr–2 wk); eccentric muscle and tendon-strengthening program (after stretching); normal sport activities (after 1 mo)	Mean 18.4 mo (after PRP treatment)	VISA-P; VAS	VAS scales: improved (G1, G2) VISA-P: less healing potential (G1); improved (G2) Overtime follow-up: both groups showed a clinically significant improvement	Statistically significant improvement

Ferrero et al. (2012) [28]	G1 and G2: local anesthesia (4 mL of 2% mepivacaine) injected; 2x PRP (6 mL) injected at a mean distance of 3 ± 0.52 wk with USG	Non-RCT	G1 (patellar tendon): 24; 14 M/10 F (4 bilateral) G2 (Achilles tendon): 24; 16 M/8 F (6 bilateral)	G1: 37.4 (21–56) yr; at least 3 mo G2: 38.6 (20–61) yr; at least 3 mo	Various treatments without any success	Minimize physical activity (after 48 hr); physiokinesitherapy gradual return to sports activities (after 2 wk)	20 d; 6 mo	VISA-P; VISA-A; US	Nonsignificant improvement (20 d FU); intratendinous vascularity increased both 20 d FU and 6 mo FU; significant improvement (6 mo FU)	Statistically significant and lasting improvement of clinical symptoms; PRP injection leads to recovery of the tendon matrix potentially helping to prevent degenerative lesions

Filardo et al. (2013) [29]	G1: 3x USG PRP injections were administered every 14 d; before the injection, 10% of CaCl_2_ was added to the PRP unit (5 mL) to activate platelets G2: no control group	Non-RCT	43/43; 42 M/1 F (11 bilateral)	30.6 ± 11.7 yr; ≥3 mo	Various treatments without any success	Rest (between 1st and 2nd injection); eccentric exercises (after 2nd injection-12 wk)	ET; 2 mo; 6 mo; up to 48.6 ± 8.1 mo	Blanzina; VISA-P; EQ-VAS; Tegner; US (26 tendons)	Good and stable results over time; significantly poorer results with a longer history of symptoms; poor results with bilateral lesions; no correlation between US and clinical findings	Good overall results for the treatment of chronic refractory patellar tendinopathy

van Ark et al. (2013) [31]	1x USG, a low concentration of platelets (433 × 10^9^/L) injected	Prospective case series	5/5; 2 M/3 F (1 bilateral)	27 (23–31) yr; ≥3 mo	Various treatments without any success	Rest, low load (0–2 wk); higher cycling intensity, home exercise program (2–4 wk); eccentric exercises, various exercises (5, 6 wk); exercises progressing to higher % 1RM, 3 × 8–15 reps., rest interval 30 sec., more muscular hypertrophy (7, 8 wk); daily eccentric training continues, advance to more sport-specific exercises (after 8 wk)	6 wk; 12 wk; 16 wk; 26 wk	VISA-P	Five of the six tendons showed an improvement of at least 30 points on the VISA-P after 26 weeks	The combination treatment reported in this study is feasible and seems to be promising for patients in the late/degenerative phase of patellar tendinopathy

Charousset et al. (2014) [32]	3x USG PRP (2 mL) injections every 1 wk	Prospective case series	28/28	27 (16–37) yr; ≥4 mo	Various treatments without any success	The rehabilitation program starting with warm-up exercises, stretching, and formal eccentric exercises on a flat board followed by progressive training such as cycling and mild exercises in the pool [71]	4 wk; 3 mo; 6 mo; 12 mo; 18 mo; 24 mo	VISA-P; VAS; Lysholm; MRI	All patients showed an improvement in all scores at the 2 yr FU and twenty-one of 28 patients returned to their presymptom sporting level at 3 mo	PRP injection allows fast recovery of athletes with patellar tendinopathy to a presymptom sporting level

Brown and Sivan (2010) [33]	1x USG PRP (3 mL) injections were administered	Case study	1/1; 1 M	36 yr; ≥9 mo	Various treatments without any success	Minimize physical activity (the few days); slow quadriceps eccentric strengthening exercises (after 2 wk)	6 wk	VISA-P; US; pain level	An improvement of at least 19 points on the VISA-P; a 50% reduction in pain; reduced thickness of the tendon	PRP injection is a safe and cost-effective treatment method for chronic patellar tendinopathy

Rowan and Drouin (2013) [34]	1x USG PRP (2 mL) injections were administered	Case study	1/1; 1 F	23 yr; ≥6 yr	Various treatments without any success	Non-weight bearing (0–2 wk); 50% weight-bearing (2-3 wk); eccentric decline-board squats and no other activity (3–7 wk); rehabilitation and aqua jogging (7–10 wk)	2 mo	US; pain level	A diagnostic ultrasound confirmed complete resolution of the defect and the patients was symptom-free.	Emerging literature on PRP appears to be promising for patellar tendinopathy.

Scollon-Grieve and Malanga (2011) [35]	1x USG PRP (5 mL) injections were administered	Case study	1/1; 1 M	18 yr; ≥1 yr	Various treatments without any success	Rest (1 wk); running, jumping, or doing resistance training (1–4 wk); progressive open chain resistance training (4–6 wk); closed chain exercises (after 6 wk)	1 mo; 2 mo	US; pain level	An estimated 90% clinical improvement in function and a complete resolution of pain (1 mo FU); full activity without pain or limitation (2 mo FU)	PRP injection is a safe and promising alternative for patients with chronic patellar tendinopathy

Mautner et al. (2013) [17]	Survey on satisfaction and functional outcome; PRP injections with USG were administered for tendinopathy refractory to conventional treatments	Retrospective; cross-sectional survey	180/27; 100 M/80 F	48 (19–73) yr; ≥6 mo	Various treatments without any success	A rehabilitation program (did not standardize the specific protocol)	15 ± 6 mo	Likert scale; VAS; functional pain; overall satisfaction	Moderate improvement in symptoms: ≥50% (patellar tendinopathy patients). Improvement in VAS: 78% (patellar tendinopathy patients)	Majority of patients reported a moderate improvement in pain symptoms

Dallaudière et al. (2014) [37]	Survey on satisfaction and functional outcome; a single intratendinous injection of PRP under US guidance	Retrospective survey	408/41	≥6 mo	Various treatments without any success	Not described	6 wk; 32 mo	WOMAC; VAS; US	Significant improvement in WOMAC score and residual US size of lesions	Intratendinous injection of PRP allows rapid tendon healing and decreases in clinical complaints in patients

G: group; USG: ultrasound-guided; MP: multiple penetration; RCT: randomized controlled trial; M: male; F: female; yr: year; mo: month; wk: week; d: day; hr: hour; VISA-P: Victorian Institute of Sports Assessment-Patellar questionnaire; Tegner: Tegner activity scale; Lysholm: Lysholm knee scoring scale; VAS: Visual Analogue Scale; SF-12: short form-12; MRI: magnetic resonance imaging; ca.: approximately; ET: end of therapy; EQ-VAS: EuroQol-Visual Analogue Scale; SF-36 questionnaires: short form-36 questionnaires (health survey score); FU: follow-up; VISA-A: Victorian Institute of Sports Assessment-Achilles questionnaire; US: ultrasound; 1RM: 1 repetition maximum; reps.: repetitions; ESWT: Extracorporeal Shock Wave Therapy; NS: ten-point numeric scale; WOMAC: Western Ontario and McMaster Universities Osteoarthritis Index.
